# Early enteral nutrition and perioperative peripheral immune-cell dynamics after gastrectomy for gastric cancer

**DOI:** 10.3389/fgene.2026.1931867

**Published:** 2026-10-22

**Authors:** Hanmei Jiang, Yajie He, Min Tian, Yingsheng Xu, Xiaohua Guo

**Affiliations:** 1 Department of Gastroenterology, Qinghai University Affiliated Hospital, Xining, Qinghai, China; 2 Department of General Surgery, Ezhou Central Hospital, Ezhou, Hubei, China; 3 Department of Outpatient, The General Hospital of Western Theater Command of Chinese People’s Liberation Army, Chengdu, Sichuan, China; 4 Department of Clinical Nutrition, Ezhou Central Hospital, Ezhou, China; 5 Department of Digestive Surgery, Honghui Hospital, Xi’an Jiaotong University, Xi’an City, Shaanxi, China

**Keywords:** CD4+/CD8+ ratio, early enteral nutrition, enhanced recovery after surgery, gastrectomy, gastric cancer, NK cells, peripheral immune cells, postoperative complications

## Abstract

**Background:**

Immune checkpoint inhibitors (ICIs) have changed advanced gastric cancer (GC) treatment, but response remains heterogeneous. Radical gastrectomy causes transient perioperative immune perturbations. We evaluated whether early enteral nutrition (EEN) was associated with preservation of peripheral immune-cell phenotypes and improved postoperative outcomes. Immunotherapy-related interpretations were exploratory; no ICI therapy or molecular pathway assays were performed.

**Methods:**

This retrospective cohort included 120 patients undergoing radical gastrectomy (January 2022-December 2023): EEN within 24 h (n = 60) or traditional delayed nutrition (TN) on postoperative day (POD) 3–5 (n = 60). The primary endpoint was the POD 7 CD4^+^/CD8^+^ ratio. Exploratory secondary endpoints included immune-cell counts, 30-day complications, nutritional and gastrointestinal recovery, hospital utilization, and quality-of-life measures. Driver mutations described baseline context only; no mutation-stratified outcome analyses were performed.

**Results:**

On POD 7, the CD4^+^/CD8^+^ ratio was higher with EEN than TN (1.42 ± 0.18 vs. 1.26 ± 0.21; mean difference, 0.16; 95% CI, 0.09-0.23; P < 0.001). CD4^+^ (512 ± 68 vs. 456 ± 72 cells/μL), CD3^+^ (1,235 ± 152 vs. 1,086 ± 168 cells/μL), and NK-cell counts (285 ± 42 vs. 248 ± 38 cells/μL) were also higher (all P < 0.001), whereas CD8^+^ counts did not differ (P = 0.743). Overall postoperative complications were lower with EEN (15.0% vs. 31.7%; risk ratio, 0.47; 95% CI, 0.23-0.96; P = 0.031), and postoperative hospital stay was shorter (10.3 ± 2.1 vs. 13.8 ± 2.9 days; P < 0.001). Secondary comparisons were exploratory and unadjusted for multiplicity.

**Conclusion:**

Perioperative EEN was associated with better preservation of selected peripheral immune-cell phenotypes, fewer postoperative complications, and faster recovery after gastrectomy for GC. CD4^+^/CD8^+^ ratio and NK-cell counts are phenotypic markers, not direct measures of *IFNG*/*IL2* or *NFKB1*/*IL6* pathway activity. Immunotherapy implications remain hypothesis-generating and require prospective validation.

## 1 Introduction

Gastric cancer remains a major contributor to the global cancer burden (Sung et al., 2026). Immune checkpoint inhibitor (ICI) therapy has substantially changed the treatment landscape of advanced GC. First-line nivolumab plus chemotherapy improves survival in appropriate patients with advanced gastric, gastro-oesophageal junction, or oesophageal adenocarcinoma (Janjigian et al., 2021), while pembrolizumab and nivolumab have demonstrated activity in previously treated disease (Fuchs et al., 2018; Kang et al., 2017). Nevertheless, treatment response remains heterogeneous. Established biomarkers such as PD-L1 combined positive score (CPS), tumor mutational burden (TMB), and microsatellite instability (MSI) provide useful information but do not capture all dimensions of antitumor immunity (Cristescu et al., 2018; Sharma and Allison, 2015). This has motivated interest in dynamic immune phenotypes that can be measured over time.

The molecular landscape of gastric adenocarcinoma is heterogeneous. The Cancer Genome Atlas (TCGA) defined Epstein-Barr virus-positive, microsatellite-unstable, genomically stable, and chromosomal-instability subtypes, with recurrent alterations involving genes such as *TP53*, *ERBB2*, and *KRAS* (Cancer Genome Atlas Research Network, 2014). These molecular features may influence tumor biology and immune context, but the present study did not measure tumor immune gene expression or perform mutation-stratified treatment-effect analyses. More broadly, immune-exclusion pathways can influence checkpoint-blockade response across tumor types, although cross-tumor generalization requires caution (Mariathasan et al., 2018). In this study, driver-mutation data were therefore used primarily to characterize baseline molecular context and group balance.

Radical gastrectomy remains a central curative treatment for resectable gastric cancer (Japanese Gastric Cancer Association, 2026). Major cancer surgery can induce transient systemic inflammation and postoperative immunosuppression, including changes in T-cell and NK-cell number or function and in circulating cytokine patterns (Yang et al., 2024; Tang et al., 2020). The peripheral CD4^+^/CD8^+^ ratio and NK-cell counts are clinically accessible phenotypic measures of immune composition. Although T-cell-inflamed states and pre-existing cytotoxic T-cell activity are relevant to checkpoint-blockade biology (Cristescu et al., 2018; Tumeh et al., 2014), peripheral cell counts should not be interpreted as direct measurements of *IFNG*/*IL2* gene-expression status or as validated predictors of ICI response in gastric cancer.

Early enteral nutrition (EEN) is consistent with enhanced-recovery principles after gastrectomy (Mortensen et al., 2014). Observational evidence supports the feasibility of early postoperative enteral feeding after major digestive surgery (Braga et al., 2002a), and current perioperative nutrition guidance favors early restoration of oral or enteral intake when clinically appropriate (Weimann et al., 2021). Meta-analytic evidence also supports early enteral feeding after intestinal surgery (Lewis et al., 2009). Immunonutrition formulations containing nutrients such as arginine and n-3 fatty acids have shown immunometabolic and clinical effects in gastrointestinal cancer surgery (Braga et al., 2002b), and a randomized trial in major upper gastrointestinal surgery reported improved clinical outcomes with EEN (Barlow et al., 2011). These observations provide a biological rationale for studying perioperative immune phenotypes, but the present cohort did not directly assess intestinal tight-junction genes, *MTOR* signaling, glutamine transport, prostaglandin production, or cytokine gene expression.

The present study therefore had three aims: (1) to test whether EEN was associated with the primary endpoint of the peripheral CD4^+^/CD8^+^ ratio on POD 7 after gastrectomy; (2) to explore additional peripheral immune-cell counts and postoperative nutritional, gastrointestinal, complication, hospitalization, quality-of-life, and satisfaction outcomes; and (3) to place these phenotypic observations in an exploratory immunotherapy-relevant context while clearly distinguishing measured clinical data from unmeasured molecular mechanisms. Driver-mutation frequencies were assessed for baseline context only, not for subgroup efficacy comparisons.

## 2 Methods

### 2.1 Study design and ethical approval

This retrospective cohort study analyzed clinical data from 120 patients with pathologically confirmed gastric adenocarcinoma who underwent radical gastrectomy at the Department of Gastroenterology, Affiliated Hospital of Qinghai University, between January 2022 and December 2023. The study protocol was approved by the institutional review board (Ethics Approval No.: 2022-GS-086) and conducted in accordance with the Declaration of Helsinki. Written informed consent was obtained from all patients or their legal representatives. Group allocation was based on institutional ERAS protocol guidelines: EEN was administered to patients meeting standardized early enteral tolerance criteria, while TN was applied when early feeding was clinically contraindicated. The protocol-based (non-randomized) nature of this allocation is a key limitation; confounding by indication cannot be fully excluded despite broadly similar measured baseline characteristics.

### 2.2 Patient selection and driver mutation characterization

Inclusion criteria: (1) age 18–75 years; (2) pathologically confirmed gastric adenocarcinoma with available driver gene mutation testing results (*TP53*, *CDH1*, *ERBB2*, *KRAS*) documenting immunological subtype context; (3) radical gastrectomy (total or subtotal + D2 lymph node dissection); (4) ASA physical status grade I–III; and (5) complete clinical, immune biomarker, and follow-up data. Exclusion criteria: emergency or palliative surgery; R1/R2 resection; severe preoperative malnutrition (weight loss >10% in 6 months, albumin <30 g/L); severe organ dysfunction; prior abdominal surgery affecting gastrointestinal function; inability to tolerate enteral nutrition; or incomplete/lost follow-up data. Driver mutation status was determined using an NGS panel (TP53, KRAS mutations), immunohistochemistry (IHC) and FISH for ERBB2/HER2, all performed on FFPE resected tumor tissue per institutional standard of care.

### 2.3 Perioperative intervention protocols

#### 2.3.1 EEN group (n = 60)

Enteral nutrition was initiated via a nasojejunal tube or jejunostomy within 24 h postoperatively, beginning with 5% glucose solution (20–30 mL/h) and transitioning progressively to a standard enteral formula. By POD 3, the target caloric intake was 25–30 kcal/kg/day and the target protein intake was 1.2–1.5 g/kg/day. The intervention was defined clinically by timing and delivery of enteral nutrition; molecular pathway activation was not measured.

#### 2.3.2 TN group (n = 60)

In the TN group, enteral nutrition was delayed until POD 3-5 following recovery of gastrointestinal function, with parenteral nutrition predominating during the early postoperative period. This group served as the clinical comparator for timing of postoperative nutritional support; no molecular effects of fasting or parenteral nutrition were directly assayed.

### 2.4 Dynamic tumor-immune biomarker assessment

The primary endpoint was the peripheral CD4^+^/CD8^+^ ratio on POD 7. Exploratory secondary immune outcomes included absolute peripheral CD3^+^, CD4^+^, CD8^+^, and NK-cell (CD56^+^) counts at the available preoperative, POD 3, and POD 7 time points. These measures were treated as phenotypic immune-cell markers and were not used as direct proxies for *IFNG*/*IL2* gene expression. Thirty-day postoperative complications were recorded using the Clavien-Dindo framework as clinical outcomes; they were not considered direct measurements or validated surrogates of *NFKB1*/*IL6* pathway activity. Driver-mutation status was used to describe cohort context only; no mutation-stratified outcome analysis was performed.

### 2.5 Supporting clinical outcome indicators

Exploratory secondary clinical outcomes included: (1) nutritional-metabolic indicators, including serum albumin, prealbumin, and hemoglobin at the available time points shown in Table 1; (2) gastrointestinal recovery indices, including time to first flatus, first defecation, resumption of oral intake, and full oral diet; (3) 30-day postoperative complications; (4) postoperative and total length of hospital stay and hospitalization costs; and (5) EORTC QLQ-C30/QLQ-STO22 and patient-satisfaction measures at the reported follow-up time points. These outcomes were interpreted clinically rather than as direct readouts of specific gene-regulatory pathways.

**TABLE 1:** Perioperative nutritional and hematologic indicators.

Nutritional indicator	Time point	EEN group	TN group
Serum albumin (g/L)	Preoperative	42.3 ± 3.1	41.8 ± 3.4
​	POD 3	36.2 ± 3.5***	33.8 ± 4.1
​	POD 7	38.5 ± 3.2***	35.8 ± 3.8
​	POD 14	40.8 ± 2.9***	37.2 ± 3.6
Prealbumin (mg/L)	Preoperative	268.5 ± 32.8	265.3 ± 35.2
​	POD 7	245.3 ± 28.6***	218.7 ± 32.4
Hemoglobin (g/L)	Preoperative	128.4 ± 15.2	126.8 ± 16.8
​	POD 7	116.8 ± 12.4***	108.2 ± 14.6

POD, postoperative day. ***P < 0.001 versus TN group for the indicated comparisons. Albumin, prealbumin, and hemoglobin were analyzed as clinical nutritional or hematologic measures, not as surrogate measurements of specific gene pathways.

### 2.6 Statistical analysis

Statistical analyses were performed using SPSS 26.0 (IBM Corporation, USA). Continuous variables are presented as mean ± SD and were compared using independent-samples t tests or Mann-Whitney U tests, as appropriate. Categorical variables are presented as frequencies and percentages and were compared using the chi-square test or Fisher exact test, as appropriate; the overall postoperative complication rate was evaluated with the Pearson chi-square test because expected cell counts were adequate. For the primary endpoint, the between-group mean difference with a 95% confidence interval (CI) is reported; for the overall postoperative complication rate, a risk ratio with 95% CI is reported. All tests were two-sided, and P < 0.05 was considered statistically significant. Longitudinal measurements are presented at their available perioperative time points. Because complete verified repeated-measures model output was not available in the analysis record, time, group, and time × group interaction statistics were not reconstructed from aggregate means and SDs and are not reported. All secondary analyses were exploratory, and no adjustment for multiple comparisons was performed; secondary P values are therefore nominal and should be interpreted cautiously.

## 3 Results

### 3.1 Baseline characteristics and driver mutation landscape

A total of 120 patients were included, with 60 patients in each group. As shown in Table 2, no statistically significant between-group differences were identified for age, sex, BMI, tumor location, TNM stage, surgical approach, operative time, intraoperative blood loss, or the reported driver-mutation frequencies (all P > 0.05). These nonsignificant baseline comparisons describe the observed sample but do not establish statistical equivalence between groups. *TP53* mutation, *ERBB2* (HER2) positivity, and *KRAS* mutation frequencies were treated as descriptive baseline characteristics and were not used to infer mutation-specific treatment effects.

**TABLE 2:** Baseline characteristics of the patients.

Characteristic	EEN Group (n = 60)	TN Group (n = 60)
Age (years)	58.3 ± 10.2	59.7 ± 9.8
Sex (Male/Female)	38/22	41/19
BMI (kg/m^2^)	22.8 ± 2.6	23.2 ± 2.4
Tumor location (Upper/Middle/Lower)	18/22/20	20/19/21
TNM stage (II/III)	35/25	33/27
Surgery type (Total/Subtotal gastrectomy)	22/38	25/35
Driver mutation status (descriptive only)		
*TP53* mutation rate	51.7% (31/60)	55.0% (33/60)
*ERBB2* (HER2) positivity rate	18.3% (11/60)	16.7% (10/60)
*KRAS* mutation rate	15.0% (9/60)	13.3% (8/60)
Operative time (min)	218 ± 38	225 ± 42
Intraoperative blood loss (mL)	186 ± 52	192 ± 48

No statistically significant between-group differences were detected for the listed baseline characteristics or driver-mutation frequencies (all P > 0.05); this should not be interpreted as proof of baseline equivalence. Mutation categories may overlap. Driver-mutation data were used for descriptive baseline context only; no mutation-stratified outcome comparisons were performed.

### 3.2 Perioperative peripheral immune-cell dynamics

As shown in Table 3 and Figures 1 and 2, several peripheral lymphocyte and NK-cell measures declined after surgery in both groups. The primary endpoint, the CD4^+^/CD8^+^ ratio on POD 7, was higher in the EEN group than in the TN group (1.42 ± 0.18 vs. 1.26 ± 0.21; mean difference, 0.16; 95% CI, 0.09-0.23; P < 0.001). At POD 3, between-group differences were also present for CD3^+^ counts (P < 0.001), CD4^+^ counts (P < 0.001), the CD4^+^/CD8^+^ ratio (P = 0.007), and NK-cell counts (P < 0.001). On POD 7, CD4^+^ counts (512 ± 68 vs. 456 ± 72 cells/μL), CD3^+^ counts (1,235 ± 152 vs. 1,086 ± 168 cells/μL), and NK-cell counts (285 ± 42 vs. 248 ± 38 cells/μL) were higher in the EEN group (all P < 0.001). In contrast, POD 7 CD8^+^ counts were similar between groups (362 ± 48 vs. 365 ± 52 cells/μL; P = 0.743), indicating that the between-group difference in the CD4^+^/CD8^+^ ratio was primarily associated with higher CD4^+^ counts rather than lower CD8^+^ counts. These variables are phenotypic immune-cell measures and should not be interpreted as direct readouts of *IFNG*/*IL2* gene expression. Longitudinal trajectories are shown descriptively because verified time × group repeated-measures statistics were not available for reporting.

**TABLE 3:** Perioperative peripheral immune-cell measures.

Immune-cell measure	Time point	EEN group	TN group
CD3^+^ count (cells/μL)	Preoperative	1,458 ± 182	1,442 ± 196
​	POD 3	1,312 ± 165***	1,195 ± 178
​	POD 7	1,235 ± 152***	1,086 ± 168
CD4^+^ count (cells/μL)	Preoperative	685 ± 82	678 ± 88
​	POD 3	598 ± 75***	542 ± 80
​	POD 7	512 ± 68***	456 ± 72
CD8^+^ count (cells/μL)	Preoperative	468 ± 58	462 ± 62
​	POD 7	362 ± 48	365 ± 52
CD4^+^/CD8^+^ ratio	Preoperative	1.46 ± 0.16	1.47 ± 0.18
​	POD 3	1.44 ± 0.17**	1.35 ± 0.19
​	POD 7	1.42 ± 0.18***	1.26 ± 0.21
NK cells (CD56^+^) (cells/μL)	Preoperative	352 ± 48	348 ± 52
​	POD 3	318 ± 45***	285 ± 42
​	POD 7	285 ± 42***	248 ± 38

POD, postoperative day. Between-group comparisons shown in Table 3 were assessed by independent-samples t tests. **P < 0.01; ***P < 0.001 versus TN group; POD 3 CD8^+^ data were not available in the analysis table and are therefore not presented. POD 7 CD8^+^ counts did not differ significantly between groups (P = 0.743). The CD4^+^/CD8^+^ ratio and NK-cell counts are phenotypic immune markers; no corresponding gene-expression assay was performed.

**FIGURE 1:**
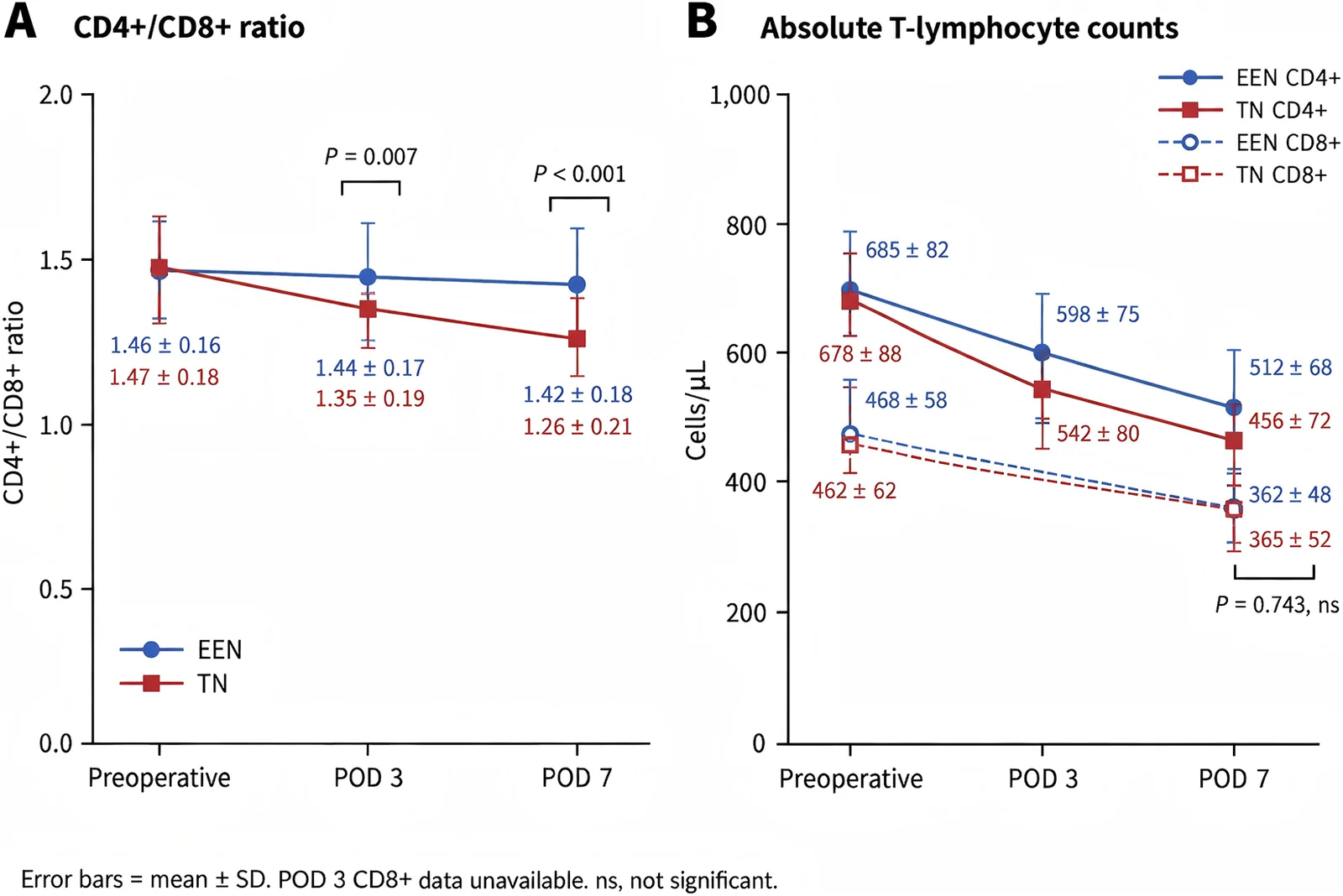
Perioperative trajectory of the peripheral CD4^+^/CD8^+^ ratio and T-lymphocyte counts. **(A)** Serial CD4^+^/CD8^+^ ratio measurements at preoperative baseline, postoperative day (POD) 3, and POD 7. The between-group difference was significant at POD 3 (P = 0.007) and POD 7 (P < 0.001). **(B)** Absolute CD4^+^ and CD8^+^ T-cell counts across the available perioperative time points. POD 3 CD8^+^ data were not available and are not shown; POD 7 CD8^+^ counts were not significantly different (P = 0.743). These measures represent peripheral immune-cell phenotypes and not direct *IFNG*/*IL2* gene-expression measurements. Error bars represent mean ± SD. **P < 0.01; ***P < 0.001 versus TN group; ns, not significant. EEN, early enteral nutrition; TN, traditional nutrition; POD, postoperative day.

**FIGURE 2:**
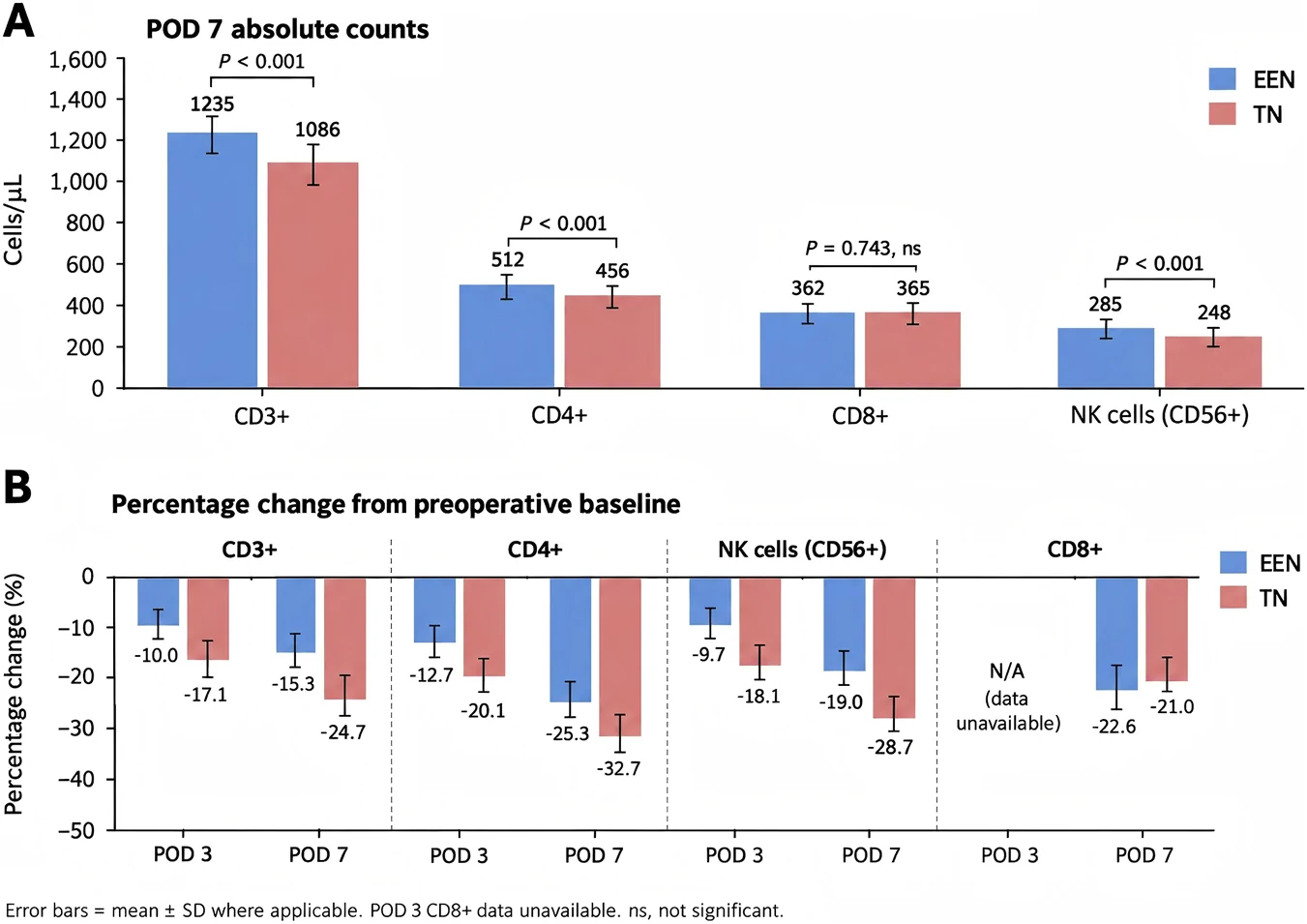
Peripheral immune-cell counts and changes from baseline. **(A)** Absolute CD3^+^, CD4^+^, CD8^+^, and NK-cell (CD56^+^) counts at POD 7. CD3^+^, CD4^+^, and NK-cell counts were higher in the EEN group (all P < 0.001), whereas CD8^+^ counts did not differ significantly between groups (P = 0.743). **(B)** Percentage changes from preoperative baseline are shown only for measures with available data at the relevant time points; POD 3 CD8^+^ data were unavailable. Error bars represent mean ± SD. The figure should not be interpreted as demonstrating direct molecular pathway activity.

### 3.3 Postoperative complications and clinical outcomes

As shown in Table 4 and Figure 3, the overall postoperative complication rate was lower in the EEN group than in the TN group (15.0% vs. 31.7%; risk ratio, 0.47; 95% CI, 0.23-0.96; Pearson χ^2^ P = 0.031). The observed risk in the EEN group was approximately 53% lower relative to the TN group. Individual complication categories were numerically less frequent in the EEN group, including anastomotic leakage (1.7% vs. 5.0%), pulmonary infection (3.3% vs. 10.0%), wound infection (3.3% vs. 8.3%), intra-abdominal abscess (1.7% vs. 5.0%), and delayed gastric emptying (5.0% vs. 11.7%). Because individual patients could experience more than one event, the category counts are not mutually exclusive; the total complication rate represents patients with at least one postoperative complication. These clinical outcomes may be compatible with a lower postoperative inflammatory burden but do not directly demonstrate activation or attenuation of *NFKB1*/*IL6* or other molecular pathways.

**TABLE 4:** Postoperative complications within 30 days.

Complication type	EEN group (n = 60)	TN group (n = 60)
Anastomotic leakage	1 (1.7%)	3 (5.0%)
Pulmonary infection	2 (3.3%)	6 (10.0%)
Wound infection	2 (3.3%)	5 (8.3%)
Intra-abdominal abscess	1 (1.7%)	3 (5.0%)
Delayed gastric emptying	3 (5.0%)	7 (11.7%)
Other complications	1 (1.7%)	2 (3.3%)
Patients with ≥1 complication	9 (15.0%)*	19 (31.7%)

*P = 0.031 versus TN group for the overall postoperative complication rate (Pearson chi-square test). Individual complication categories were not mutually exclusive; the total represents patients with at least one postoperative complication. Complication patterns are clinical outcomes and are not direct molecular pathway measurements.

**FIGURE 3:**
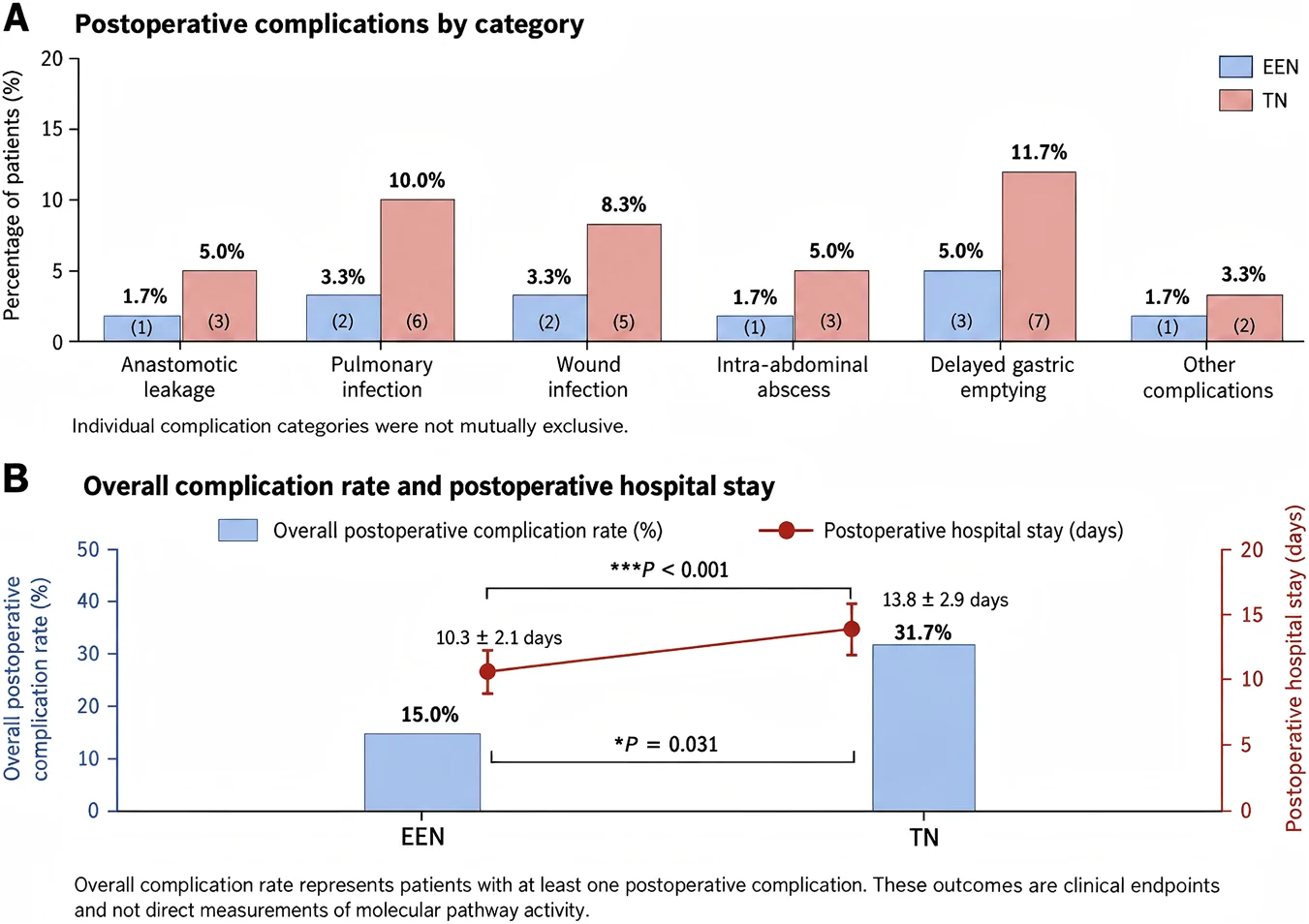
Postoperative complication profile and hospital stay. **(A)** Individual postoperative complication categories in the EEN and TN groups. Individual categories were not mutually exclusive. **(B)** Overall postoperative complication rate (bar chart, left y-axis) and postoperative hospital stay (line graph, right y-axis). The EEN group had a lower overall complication rate (15.0% vs. 31.7%, *P = 0.031; Pearson chi-square test) and a shorter postoperative hospital stay (10.3 ± 2.1 vs. 13.8 ± 2.9 days, ***P < 0.001). These outcomes are clinical endpoints and should not be interpreted as direct measurements of *NFKB1*/*IL6* pathway activity.

### 3.4 Nutritional-metabolic recovery

Table 1 summarizes nutritional-metabolic recovery. On POD 7, the EEN group had higher serum albumin (38.5 ± 3.2 vs. 35.8 ± 3.8 g/L, P < 0.001) and prealbumin (245.3 ± 28.6 vs. 218.7 ± 32.4 mg/L, P < 0.001) than the TN group. Hemoglobin on POD 7 was also higher in the EEN group (116.8 ± 12.4 vs. 108.2 ± 14.6 g/L, P < 0.001). These findings support better biochemical nutritional recovery with EEN; they do not directly establish *MTOR* pathway activation or immune-cell metabolic signaling.

### 3.5 Gastrointestinal function recovery

Table 5 shows faster gastrointestinal recovery in the EEN group. Time to first flatus was shorter (2.8 ± 0.6 vs. 3.9 ± 0.8 days, P < 0.001), as was time to first defecation (3.5 ± 0.7 vs. 5.2 ± 1.1 days, P < 0.001). Resumption of oral intake (4.2 ± 0.8 vs. 6.3 ± 1.2 days) and achievement of a full oral diet (7.1 ± 1.3 vs. 9.8 ± 1.8 days) were also earlier in the EEN group (both P < 0.001). These results are consistent with the clinical rationale for early postoperative enteral feeding (Braga et al., 2002a; Lewis et al., 2009; Barlow et al., 2011) but do not directly demonstrate activation of specific motility or epithelial signaling genes.

**TABLE 5:** Postoperative gastrointestinal recovery.

Gastrointestinal recovery indicator	EEN Group (n = 60)	TN Group (n = 60)
Time to first flatus, days	2.8 ± 0.6***	3.9 ± 0.8
Time to first defecation, days	3.5 ± 0.7***	5.2 ± 1.1
Time to resumption of oral intake, days	4.2 ± 0.8***	6.3 ± 1.2
Time to full oral diet, days	7.1 ± 1.3***	9.8 ± 1.8

***P < 0.001 versus TN group. Gastrointestinal recovery measures are clinical endpoints; no motility-gene or intestinal-barrier gene-expression assays were performed.

### 3.6 Hospitalization and cost outcomes

As shown in Table 6, postoperative hospital stay was shorter in the EEN group than in the TN group (10.3 ± 2.1 vs. 13.8 ± 2.9 days, P < 0.001), and total hospital stay was also shorter (15.8 ± 2.6 vs. 19.2 ± 3.4 days, P < 0.001). Although nutritional support costs were higher with EEN (CNY 4,850 ± 680 vs. 3,620 ± 520, P < 0.001), total hospitalization costs were lower (CNY 52,380 ± 8,250 vs. 61,240 ± 9,850, P < 0.001). These data indicate a net reduction in overall hospitalization resource use in the EEN group.

**TABLE 6:** Hospital stay and costs.

Clinical outcome indicator	EEN group (n = 60)	TN group (n = 60)
Postoperative hospital stay (days)	10.3 ± 2.1***	13.8 ± 2.9
Total hospital stay (days)	15.8 ± 2.6***	19.2 ± 3.4
Nutritional support cost (CNY)	4,850 ± 680***	3,620 ± 520
Total hospitalization cost (CNY)	52,380 ± 8,250***	61,240 ± 9,850

***P < 0.001 versus TN group.

### 3.7 Quality of life and patient satisfaction

EORTC QLQ-C30 global health status scores were higher in the EEN group at discharge (65.8 ± 8.2 vs. 58.3 ± 9.6, P < 0.001) and at 1 month postoperatively (78.5 ± 7.4 vs. 72.1 ± 8.8, P < 0.001). Patient satisfaction was also higher for nutritional-support management (4.62 ± 0.38 vs. 3.95 ± 0.62, P < 0.001) and perceived recovery speed (4.55 ± 0.44 vs. 4.08 ± 0.58, P < 0.001). Because these were exploratory secondary outcomes and no multiplicity adjustment was applied, they should be interpreted cautiously.

## 4 Discussion

This retrospective cohort study provides preliminary observational evidence that perioperative EEN is associated with a higher POD 7 peripheral CD4^+^/CD8^+^ ratio, higher POD 7 CD3^+^, CD4^+^, and NK-cell counts, fewer overall postoperative complications, and faster clinical recovery after gastrectomy for GC. POD 7 CD8^+^ counts did not differ significantly between groups. Importantly, no gene-expression, cytokine-protein, or signaling-pathway assays were performed, and no patient received ICI therapy as part of this study. Accordingly, mechanistic and immunotherapy-related interpretations are hypothesis-generating rather than demonstrated findings.

### 4.1 Peripheral CD4^+^/CD8^+^ ratio as a phenotypic immune marker

The peripheral CD4^+^/CD8^+^ ratio is a readily measurable indicator of T-cell composition, but it is not a direct assay of *IFNG* or *IL2* expression. Tumeh et al. showed that pre-existing CD8^+^ T-cell immunity within the tumor microenvironment is relevant to anti-PD-1 response in melanoma (Tumeh et al., 2014), and pan-tumor analyses have linked T-cell-inflamed biology to checkpoint-blockade outcomes (Cristescu et al., 2018). These observations support a broad biological rationale for studying T-cell phenotypes, but they do not validate the peripheral CD4^+^/CD8^+^ ratio as a predictive biomarker in postoperative gastric cancer. In our cohort, the POD 7 ratio was higher with EEN (1.42 ± 0.18 vs. 1.26 ± 0.21; P < 0.001), with a mean difference of 0.16 (95% CI, 0.09–0.23). Because POD 7 CD8^+^ counts were similar between groups, this difference was primarily associated with higher CD4^+^ counts. The finding should therefore be interpreted as better preservation of peripheral T-cell balance, not as direct evidence of enhanced ICI candidacy or *IFNG*/*IL2* pathway activity.

### 4.2 NK-cell preservation and potential relevance to immune competence

NK-cell (CD56^+^) counts were higher in the EEN group on POD 7 (285 ± 42 vs. 248 ± 38 cells/μL, P < 0.001). NK cells are important components of innate antitumor immunity, and interactions between innate and adaptive immune responses are relevant to cancer immunology (Sharma and Allison, 2015). However, the present study measured cell counts only; it did not assess NK-cell cytotoxicity, *IFNG* production, perforin/granzyme expression, *MTOR* signaling, or nutrient-transport pathways. Consequently, the observed difference should be interpreted as preservation of a peripheral immune-cell phenotype. Whether this phenotype predicts subsequent ICI response in gastric cancer requires prospective testing.

### 4.3 Postoperative complications and inflammatory-pathway interpretation

The EEN group had a lower overall postoperative complication rate (15.0% vs. 31.7%; risk ratio, 0.47; 95% CI, 0.23-0.96; Pearson χ^2^ P = 0.031). Surgical stress is known to produce systemic inflammatory and immunosuppressive responses (Yang et al., 2024; Tang et al., 2020), so a lower complication burden may be clinically consistent with improved postoperative recovery. It cannot, however, be considered a validated surrogate for *NFKB1* or *IL6* activation. Likewise, Mariathasan et al. demonstrated a TGF-β-associated T-cell exclusion mechanism in urothelial carcinoma (Mariathasan et al., 2018), not an *NFKB1*/*IL6* mechanism in gastric cancer. These pathways should therefore remain distinct. In the present study, any connection between EEN, intestinal-barrier biology, *NFKB1*/*IL6* signaling, or later ICI efficacy is a literature-based hypothesis that was not directly tested.

### 4.4 Driver-mutation context and limits of subgroup interpretation

No statistically significant between-group differences were detected in *TP53* mutation, *ERBB2* (HER2) positivity, or *KRAS* mutation frequencies; however, nonsignificant baseline tests do not establish molecular equivalence between groups. No formal mutation-stratified outcome analyses were performed. The *ERBB2*-positive and *KRAS*-mutant subsets were small, and the study was not powered to estimate subgroup-specific treatment effects. Accordingly, mutation-subgroup consistency of the EEN association cannot be inferred from these data. Driver-mutation information should be interpreted as descriptive molecular context only. Mutation-stratified hypotheses regarding perioperative immune phenotypes and future immunotherapy outcomes require adequately powered prospective studies.

### 4.5 Hypothesis-generating relevance to future immunotherapy studies

The present findings support EEN as a perioperative nutritional strategy associated with better preservation of selected peripheral immune-cell phenotypes and improved postoperative recovery. They do not establish EEN as a pre-ICI optimization intervention or demonstrate that patients with higher postoperative CD4^+^/CD8^+^ ratios or NK-cell counts will respond better to checkpoint blockade. Serial peripheral immune phenotyping before and after surgery may be useful in future prospective studies that also include tumor-based immune profiling, direct molecular assays, and actual ICI treatment outcomes. Such studies will be required before these markers can be used for immunotherapy stratification or treatment decisions.

### 4.6 Limitations and future research priorities

This study has several important limitations. First, its retrospective single-center design creates potential selection bias and residual confounding, including confounding by indication because nutritional allocation was not randomized. Second, the study measured peripheral immune-cell counts but did not directly characterize the tumor microenvironment or measure gene expression, cytokine proteins, or signaling-pathway activity. Third, no patients received ICI therapy in this cohort, so any relationship between perioperative immune phenotypes and immunotherapy response is inferential. Fourth, no formal driver-mutation subgroup outcome analyses were performed, and the *ERBB2*-positive and *KRAS*-mutant subsets were too small for reliable treatment-effect estimation. Fifth, POD 3 CD8^+^ data were unavailable in the presented analysis, and complete verified repeated-measures model output was not available; therefore, time × group interaction statistics could not be reported. Sixth, the 1-month follow-up period precludes assessment of long-term immune recovery and oncologic outcomes.

Future research should prioritize prospective multicenter studies that: (1) test EEN using randomized or otherwise rigorous comparative designs; (2) collect complete longitudinal immune-cell data with prespecified mixed-effects or repeated-measures analyses; (3) integrate direct molecular measurements such as cytokine assays, transcriptomics, or spatial immune profiling; (4) include adequately powered mutation-defined analyses where clinically justified; and (5) evaluate actual ICI treatment response, survival, and toxicity outcomes. These steps are necessary to determine whether perioperative peripheral immune phenotypes have clinically useful predictive value beyond their role as markers of postoperative immune recovery.

## 5 Conclusion

This retrospective single-center cohort study found that perioperative EEN was associated with a higher peripheral CD4^+^/CD8^+^ ratio and higher CD3^+^, CD4^+^, and NK-cell counts on POD 7, a lower overall postoperative complication rate, faster gastrointestinal and nutritional recovery, and shorter hospital stay after gastrectomy for gastric cancer. POD 7 CD8^+^ counts did not differ significantly between groups. These immune-cell measures are phenotypic and do not directly demonstrate *IFNG*/*IL2*, *NFKB1*/*IL6*, *MTOR*, or other gene-pathway activity. No ICI therapy was administered, and no driver-mutation subgroup outcome analyses were performed. The immunotherapy relevance of perioperative immune-cell preservation therefore remains hypothesis-generating and should be evaluated in prospective studies with complete longitudinal statistics, direct molecular endpoints, and actual immunotherapy outcomes.

## Data Availability

The datasets presented in this study can be found in online repositories. The names of the repository/repositories and accession number(s) can be found in the article/supplementary material.
